# Effect of margin designs and loading conditions on the stress distribution of endocrowns: a finite element analysis

**DOI:** 10.1186/s12903-024-04422-3

**Published:** 2024-06-05

**Authors:** Baijin Zeng, Bin Luo, Jiangqi Hu, Dan Meng, Jiebing Zhang, Xu Cao, Qingsong Jiang

**Affiliations:** Department of Prosthodontics, Beijing Stomatological Hospital, School of Stomatology, Capital Medical University, No. 4 Tiantanxili, Dongcheng District, Beijing, 100050 China

**Keywords:** Endocrown, Margin design, Loading condition, Finite element analysis, The maximum von Mises stress

## Abstract

**Background:**

Margin designs and loading conditions can impact the mechanical characteristics and survival of endocrowns. Analyzing the stress distribution of endocrowns with various margin designs and loading conditions can provide evidence for their clinical application.

**Methods:**

Three finite element analysis models were established based on the margin designs: endocrown with a butt-joint type margin (E0), endocrown with a 90° shoulder (E90), and endocrown with a 135° shoulder (E135). The E0 group involved lowering the occlusal surface and preparing the pulp chamber. The E90 group created a 90° shoulder on the margin of model E0, measuring 1.5 mm high and 1 mm wide. The E135 group featured a 135° shoulder. The solids of the models were in fixed contact with each other, and the materials of tooth tissue and restoration were uniform, continuous, isotropic linear elasticity. Nine static loads were applied, with a total load of 225 N, and the maximum von Mises stresses and stress distribution were calculated for teeth and endocrowns with different margin designs.

**Results:**

Compared the stresses of different models under the same loading condition. In endocrowns, when the loading points were concentrated on the buccal side, the maximum von Mises stresses were E0 = E90 = E135, and when there was a lingual loading, they were E0 < E90 = E135. In enamel, the maximum von Mises stresses under all loading conditions were E0 > E90 > E135. In dentin, the maximum von Mises stresses of the three models were basically similar except for load2, load5 and load9. Compare the stresses of the same model under different loading conditions. In endocrowns, stresses were higher when lingual loading was present. In enamel and dentin, stresses were higher when loaded obliquely or unevenly. The stresses in the endocrowns were concentrated in the loading area. In enamel, stress concentration occurred at the cementoenamel junction. In particular, E90 and E135 also experienced stress concentration at the shoulder. In dentin, the stresses were mainly concentrated in the upper section of the tooth root.

**Conclusion:**

Stress distribution is similar among the three margin designs of endocrowns, but the shoulder-type designs, especially the 135° shoulder, exhibit reduced stress concentration.

## Background

Due to caries, trauma, and cavity preparation, the resistance of teeth will be greatly reduced, and the formation of pulp chamber pathways and root canal pathways during root canal treatment will further damages the tooth structure [1, 2]. The mandibular first molar is the earliest permanent tooth to erupt in the oral cavity, and its occlusal anatomy is relatively complex. It is more likely to be damaged by caries and external forces [3]. For small tooth defects, direct fillings, inlays or partial crowns can be used, while for large tooth defects, full crowns or post-core retained crowns are the choice of most clinicians [4]. For molars that have undergone root canal treatment and have large defects, if the crown is not protected at a later stage, even normal functional forces will cause cusp damage or even root fracture under long-term loading cycles [5].

When full crowns are used to repair posterior tooth defects, the amount of preparation can reach 67.5–75.6% of the tooth tissue, and the loss of tooth tissue is relatively large [6, 7]. With the rise of the minimally invasive concept, clinicians are actively looking for restorative methods that can preserve more tooth tissue. With the advancement of bonding technology and crown materials, the application of all-ceramic endocrowns have been promoted [8]. The endocrown is a restoration that is fixed in the pulp cavity of the posterior tooth. It usually consists of an annular butt-joint type margin and a central retention cavity embedded in the pulp cavity. It is an integrated core-crown structure [9]. For teeth that have undergone root canal treatment and have extensive coronal hard tissue defects, the fracture resistance of endocrowns are better than that of traditional post-core retained crown [10]. In addition, the minimally invasive principle of tooth preparation for endocrowns is in line with the concepts of modern prosthodontics, which has attracted increasing attention [11, 12].

The basic design of endocrowns is guided by the amount of remaining tooth structure after removing all defective and unsupported tooth tissue, but there is no consensus on the design of the margin. Studies have shown that endocrowns with different margin designs have a direct effect on the marginal strength and stress distribution of teeth and restorations, and therefore on the ultimate service life of the restorations. Endocrowns margin designs fall into two main categories. One is the butt-joint type, where the margin directly forms a platform for bonding with the tooth tissue. It has the most clinical applications and the most corresponding laboratory research. The other is a shoulder type, where based on the butt-joint type, a certain ferrule is designed to form a hoop effect similar to a full crown. The shoulder type can be divided into 90° and 135° according to the angle of the shoulder [13]. There is no consensus on which margin design is better for endocrowns. Tooth structure, position, loading conditions, etc. may affect the impact of margin design on endocrowns. Guo et al. established different margin design models for all-ceramic endocrowns of the mandibular first premolar, analyzed the stress distribution of different margin shapes on the tooth tissue and restorations, and found that the stresses of the butt-joint margin were the lowest, the stress distribution of the 90° shoulder were more uniform than that of the 135° shoulder [14]. Taha et al. compared CAD/CAM polymer infiltrated ceramic endocrowns with two margin designs: butt-joint type and 90° shoulder type, the results showed that the shoulder type design had higher flexural strength [15].

In addition to margin designs, loading conditions can also affect the performance of endocrowns. When studying crowns, the loading methods commonly used by researchers mainly include two categories, vertical uniform loading and angular uniform loading [16]. Zheng et al. designed four endocrowns with different margin: flat butt joint type, 20° bevel type, 90° shoulder type and anatomic type, and found that the margin designs can affect the stress values and distribution of endocrowns under sliding vertical loading [17]. However, the force transmitted by teeth during the actual chewing process is more complex, especially when there are abnormal tooth arrangements, irregular occlusal surface shapes, abnormal occlusal relationships, etc., the teeth may be subjected to abnormal occlusal forces from any position and any angle [18, 19]. Therefore, it is important to observe the performance of restorations under more loading conditions to more comprehensively evaluate the advantages and disadvantages of different restoration designs. In this study, more different positions, different ranges, different directions and extreme loading conditions were designed to compare the stress distribution of endocrowns with different margin designs.

Methods to study the performance of restorations include finite element analysis (FEA), in vitro experiments and clinical trials, etc. FEA is a technology that simplifies complex objects into structures composed of several basic units and analyzes the force and deformation of the objects by establishing mathematical models. This method has the advantage of controlling a single variable and limiting other variable factors, and overcomes the limitation in physical research that it is difficult to have two isolated teeth that are identical in geometric form and tissue structure [20]. The FEA model can be repeatedly loaded with loads in different directions, ranges and positions. Repeated loading can effectively ensure the accuracy of the model. The stress distribution of each part of the model can be observed from any angle, which helps to analyze the stress distribution more intuitively. Therefore, FEA is a common method used to simulate and analyze the effect of restoration designs on the stresses of teeth and restorations [21].

This study aims to establish endocrown models of mandibular first molars with three different margin designs.: butt-joint type, 90° shoulder type and 135° shoulder type. FEA was performed to investigate the stress magnitude and distribution of teeth and restorations under 9 different loading conditions, providing more references for the theoretical research and clinical application of endocrowns. The null hypothesis is that there is no difference in the stress values and distribution of endocrowns with different margin designs under different loading conditions.

## Methods

### Sample selection

After obtained consent from the patient, a clinically extracted adult right mandibular first molar was selected. The collected teeth were those that had to be extracted due to severe periodontal disease, and there was no additional damage due to the needs of research. Selection criteria: good appearance, no caries, no defects, no fillings or restorations, and the size was close to the average of the adult mandibular first molars. A 10x magnifying glass was used to check that there was no crack on the root of the tooth. After cleaned the tooth surface and removed periodontal ligament, calculus and other attachments on the tooth surface, stored it in 1% chloramine T solution at 4 °C for use [14].

### Tooth scanning and reconstruction of 3D digital models

The RS-9 micro-CT (GE Healthcare, USA) was used to scan the teeth, with the long axis of the teeth parallel to the examination table and perpendicular to the scanning plane. The scan thickness was 0.019 mm, and the DICOM format data of the tooth were obtained. Mimics16.0 software (Materialise, Leuven, Belgium) readed the data, discriminated enamel, dentin, and pulp chamber through threshold analysis and adjustment processing, calculated and generated a tooth tissue point cloud model of the mandibular first molar. Geomagics 2021 software was used to smooth the surface, generated a fitting surface and solidify it.

### Creation of three-dimensional digital models of endocrowns with different margin designs

**Fig. 1:**
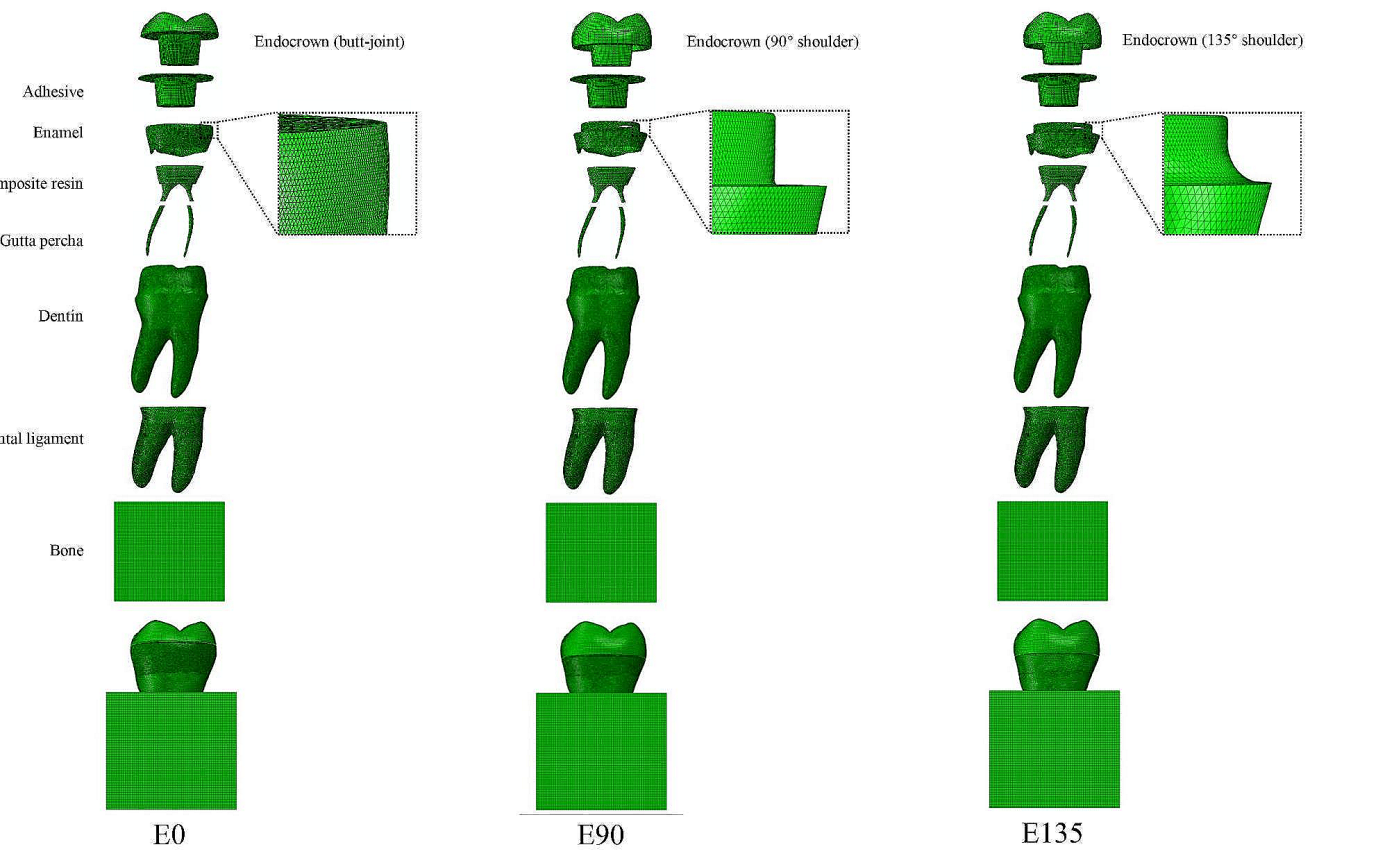
Finite element analysis model of each group. butt-joint type (E0), 90° shoulder (E90); 135° shoulder (E135)

Based on the established three-dimensional digital model of the mandibular first molar, SolidWorks2021 software (SolidWorks Corporation, Waltham, MA, USA) was used to simulate tooth preparation and root canal treatment, and establish periodontal ligament (PDL; thickness: 0.2 mm) and alveolar bone models. Following the specifications of the nickel-titanium system commonly used in clinical root canal treatment, a #25/0.06 taper mesiodistal root canal was produced, with a root apex of 0.25 mm and a root canal orifice of 1.0 mm. The root canal was filled with gutta-percha to 2 mm from the root canal orifice, sealed the upper 2 mm of the root canal orifice with resin, and flattened the bottom of the pulp chamber. The thickness of the adhesive was 50 μm. Three models were established based on the endocrowns margin designs, E0 group: butt-joint type, E90 group: 90° shoulder; E135 group: 135° shoulder. The E0 group only lowered the occlusal surface and prepared the pulp chamber. The occlusal surface thickness was reduced by 1.5 mm, the pulp chamber wall had no undercuts, the point-line angles were rounded, and the pulp chamber wall abducted 5°. The E90 group created a 90° shoulder with dimensions of 1.5 mm in height and 1 mm in width on the margin of model E0. The E135 group featured a 135° shoulder with dimensions of 1.5 mm in height and 1 mm in width on the margin of model E0 (Fig. 1).

### Experimental condition assumptions and boundary condition settings

The solids of the models were in fixed contact with each other, and the materials of tooth tissue and restorations were uniform, continuous, isotropic linear elasticity. All models were set to be fixed on the alveolar bone bottom surface. The Young’s modulus and Poisson’s ratio of each part were shown in Table 1.

**Table 1:** Mechanical properties of the materials used in FEA

	Young’s modulus (GPa)	Poisson ratio (µ)	References
Enamel	84.1	0.33	[22]
Dentin	18.6	0.31	[23]
Cortical bone	13.7	0.3	[23]
Spongious bone	1.37	0.3	[23]
Adhesive	8.3	0.35	[23]
Gutta percha	0.00069	0.45	[24]
Composite resin	15.8	0.24	[25]
Periodontal ligament	0.0689	0.45	[23]
Endocrown	95	0.25	[26]

### Loading conditions

**Fig. 2:**
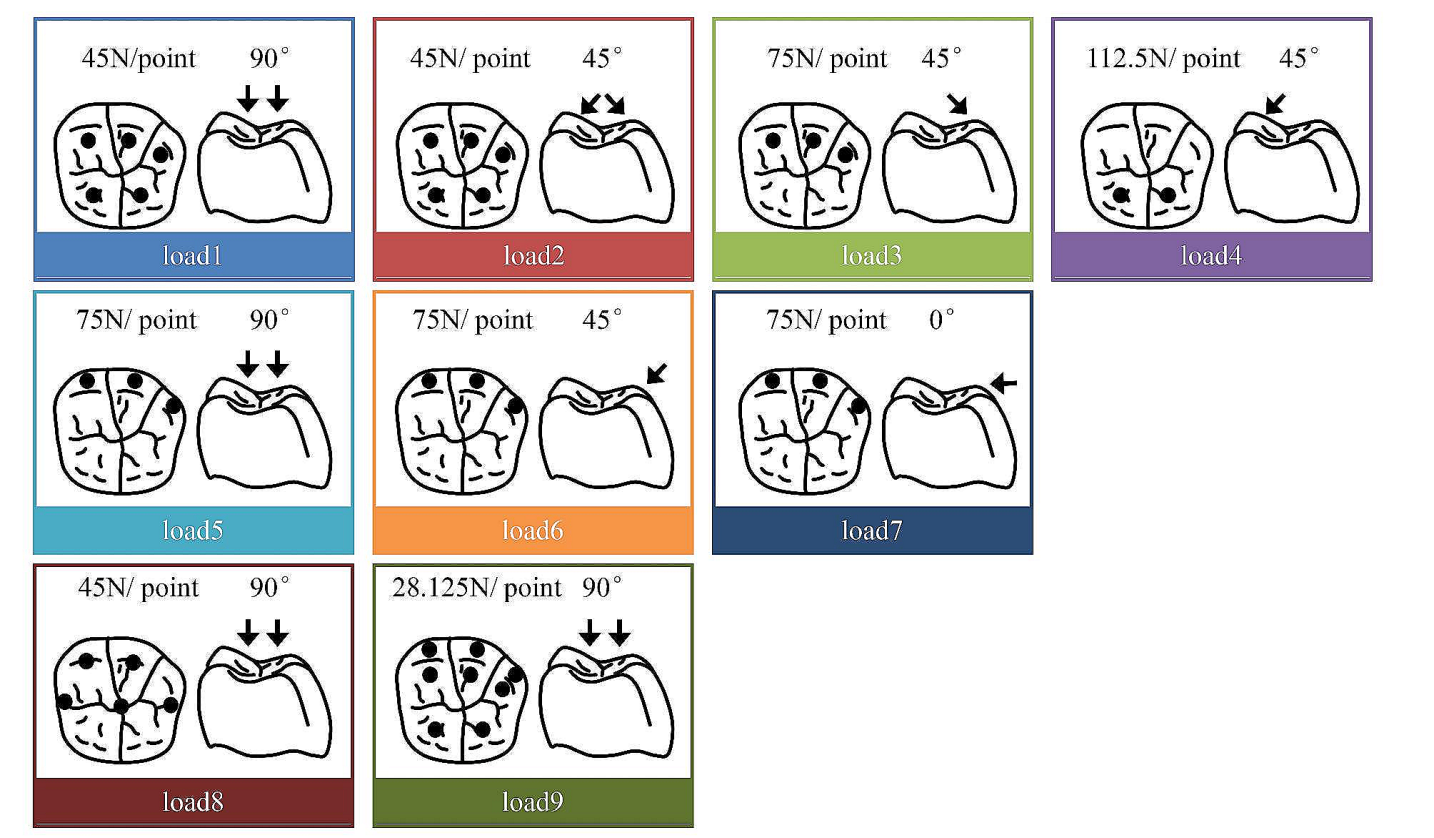
Schematic diagram of loading conditions

Nine static loads were applied under central occlusion, with a total load of 225 N (Fig. 2). Load1: 3 points on the lingual slope of the buccal cusp and 2 points on the buccal slope of the lingual cusp, 45 N per point, vertical loading. Load2: 3 points on the lingual slope of the buccal cusp and 2 points on the buccal slope of the lingual cusp, 45 N per point, 45° loading. Load3: 3 points on the lingual slope of the buccal cusp, 75 N per point, 45° loading. Load4: 2 points on the buccal slope of the lingual cusp, 112.5 N per point, 45° loading. Load5: 3 points on the buccal side of the mesial buccal cusp, distal buccal cusp and distal cusp, 75 N per point, vertical loading. Load6: 3 points on the buccal side of the mesial buccal cusp, distal buccal cusp and distal cusp, 75 N per point, 45° loading. Load7: 3 points on the buccal side of the mesial buccal cusp, distal buccal cusp and distal cusp, 75 N per point, 90° loading. Load8: 5 points on the mesial buccal cusp, distal buccal cusp, mesial marginal ridge, central fovea, and distal marginal ridge, 45 N per point, vertical loading. Load9: 8 points on the buccal and lingual slopes of the mesial buccal cusp, distal buccal cusp and distal cusp, and the buccal slope of the lingual cusp, 28.125 N per point, vertical loading. The area of each loading point was 0.5 mm^2^.

### Analysis indicators

FEA was an effective method to analyze the stress distribution of restorations. Commonly used parameters include maximum principal stresses and maximum von Mises stresses. The maximum principal stresses reflected the maximum stresses in a single direction, and the maximum von Mises stresses were the synthesis of various stresses in the model to reflect the overall stresses at the stressed area [27]. In this study, the maximum von Mises stresses and stress distribution of teeth and endocrowns with different margin designs under different loading conditions were calculated.

## Results

### Influence of different margin designs on the maximum Von Mises stresses

**Fig. 3:**
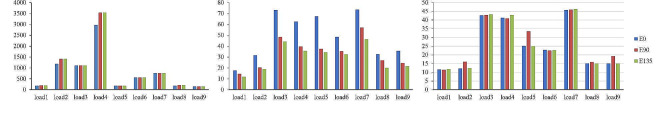
The maximum von Mises stresses of three margin design models (comparing the effects of different margin designs). The maximum von Mises stresses of endocrowns were E0 = E90 = E135 under load3, load5, load6, load7 and load9, E0< E90 = E135 under load1, load2, load4 and load8. The enamel were E0 > E90 > E135 under load1-9. The dentin were E0 = E90 = E135 under load1, load3, load4, load6, load7 and load8, E90 > E0 = E135 under load2, load5 and load9

By comparing the stresses ​​of different models under the same loading condition, the influence of different margin designs on the maximum von Mises stresses were analyzed (Table 2; Fig. 3). The maximum von Mises stresses of endocrowns were E0 = E90 = E135 under the loading conditions of load3, load5, load6, load7 and load9, E0< E90 = E135 under the loading conditions of load1, load2, load4 and load8, the maximum von Mises stresses of E0 were less than that of E90 and E135. The maximum von Mises stresses of enamel were shown as E0 > E90 > E135 under load1-9 loading conditions. The maximum von Mises stresses of E90 and E135 were similar, and E0 were significantly higher than E90 and E135. The maximum von Mises stresses of dentin were E0 = E90 = E135 under the loading conditions of load1, load3, load4, load6, load7 and load8, E90 > E0 = E135 under the loading conditions of load2, load5 and load9, the maximum von Mises stresses of E90 were higher than that of E0 and E135.

**Table 2:** The maximum von Mises stresses (MPa) for three margin design models (comparing the effects of different margin designs)

	E0	E90	E135		
	Endocrowns				
load1	189.6	197.4	197.4	E0< E90 = E135	
load2	1192.4	1418.5	1418.5	E0< E90 = E135	
load3	1123.1	1123.1	1123.1	E0 = E90 = E135	
load4	2984.5	3545.7	3545.8	E0< E90 = E135	
load5	183.4	183.3	183.3	E0 = E90 = E135	
load6	565.0	564.4	564.5	E0 = E90 = E135	
load7	770.5	769.9	770.0	E0 = E90 = E135	
load8	180.1	212.2	212.2	E0< E90 = E135	
load9	152.8	152.7	152.7	E0 = E90 = E135	

	Enamel				
load1	17.7	14.6	11.9	E0> E90> E135	
load2	31.6	20.4	19.1	E0> E90> E135	
load3	73.4	48.4	44.2	E0> E90> E135	
load4	62.6	39.6	35.7	E0> E90> E135	
load5	67.5	37.7	34.5	E0> E90> E135	
load6	48.6	35.4	32.5	E0> E90> E135	
load7	73.4	57.0	46.4	E0> E90> E135	
load8	32.7	270	20.3	E0> E90> E135	
load9	35.9	24.6	21.6	E0> E90> E135	

	Dentin				
load1	11.6	11.5	11.9	E0 = E90 = E135	
load2	12.2	16.2	12.5	E90> E0 = E135	
load3	42.7	42.9	43.3	E0 = E90 = E135	
load4	41.3	40.9	42.9	E0 = E90 = E135	
load5	25.2	33.5	25.1	E90> E0 = E135	
load6	22.8	22.4	22.7	E0 = E90 = E135	
load7	45.8	460	46.4	E0 = E90 = E135	
load8	15.1	15.9	15.0	E0 = E90 = E135	
load9	15.0	19.3	15.1	E90> E0 = E135	

### Influence of different loading conditions on the maximum Von Mises stresses

**Fig. 4:**
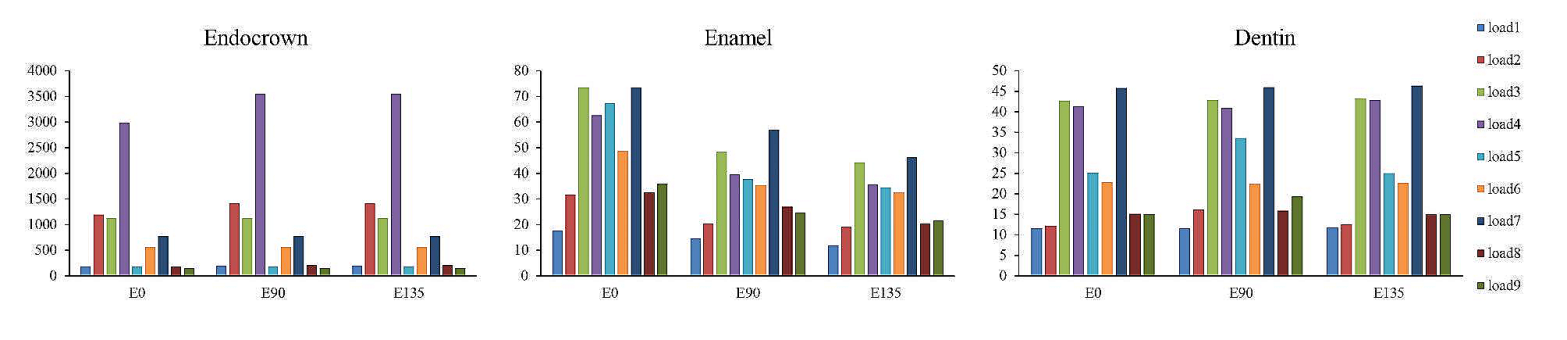
The maximum von Mises stresses of three margin design models (comparing the effects of different loading conditions). The maximum von Mises stresses of endocrowns were load4 > 3 > 2 > 7 > 6 > 1 > 5 > 8 > 9 in the E0 model, load4 > 2 > 3 > 7 > 6 > 8 > 1 > 5 > 9 in the E90 model, and load4 > 2 > 3 > 7 > 6 > 8 > 1 > 5 > 9 in the E135 model. The enamel were load7 > 3 > 5 > 4 > 6 > 9 > 8 > 2 > 1 in the E0 model, load7 > 3 > 4 > 5 > 6 > 8 > 9 > 2 > 1 in the E90 model, and load7 > 3 > 4 > 5 > 6 > 9 > 8 > 2 > 1 in the E135 model. The dentin were load7 > 3 > 4 > 5 > 6 > 8 = 9 > 2 > 1 in the E0 model, load7 > 3 > 4 > 5 > 6 > 9 > 8 > 2 > 1 in the E90 model, and load7 > 3 > 4 > 5 > 6 > 8 = 9 > 2 > 1 in the E135 model

By comparing the stresses under different loading conditions of the same model, the impact of different loading conditions on the maximum von Mises stresses were analyzed (Table 3; Fig. 4). The maximum von Mises stresses of endocrowns were load4 > 3 > 2 > 7 > 6 > 1 > 5 > 8 > 9 in the E0 model, load4 > 2 > 3 > 7 > 6 > 8 > 1 > 5 > 9 in the E90 model, and load4 > 2 > 3 > 7 > 6 > 8 > 1 > 5 > 9 in the E135 model. All three models showed that the stresses were the highest under the loading condition of load4, followed by load2, load3, load7 and load6. The stresses were less under load1, load5, load8 and load9 loading conditions. The maximum von Mises stresses of enamel were load7 > 3 > 5 > 4 > 6 > 9 > 8 > 2 > 1 in the E0 model, load7 > 3 > 4 > 5 > 6 > 8 > 9 > 2 > 1 in the E90 model, and load7 > 3 > 4 > 5 > 6 > 9 > 8 > 2 > 1 in the E135 model. The three models all showed that the stresses were higher under the loading conditions of load7, load3, load4, load5 and load6, followed by load8, load9 and load2, the stresses were the least under the loading condition of load1. The maximum von Mises stresses of dentin were load7 > 3 > 4 > 5 > 6 > 8 = 9 > 2 > 1 in the E0 model, load7 > 3 > 4 > 5 > 6 > 9 > 8 > 2 > 1 in the E90 model, and load7 > 3 > 4 > 5 > 6 > 8 = 9 > 2 > 1 in the E135 model. The three models all showed that the stresses were higher under the loading conditions of load7, load3, load4, load5 and load6, followed by load8, load9 and load2, the stresses were the least under the loading condition of load1, which were the same as the enamel stress result.

**Table 3:** The maximum von Mises stresses (MPa) of three margin design models (comparing the effects of different loading conditions)

	load1	load2	load3	load4	load5	load6	load7	load8	load9	
Endocrowns										
E0	189.6	1192.4	1123.1	2984.4	183.4	565.0	770.5	180.1	152.8	load4>3>2>7>6>1>5>8>9
E90	197.4	1418.5	1123.1	3545.7	183.3	564.4	769.9	212.2	152.7	load4>2>3>7>6>8>1>5>9
E135	197.4	1418.5	1123.1	3545.8	183.3	564.4	770.0	212.1	152.7	load4>2>3>7>6>8>1>5>9

Enamel										
E0	17.7	31.6	73.4	62.6	67.5	48.6	73.4	32.7	35.9	load7>3>5>4>6>9>8>2>1
E90	14.6	20.4	48.4	39.6	37.7	35.4	570	270	24.6	load7>3>4>5>6>8>9>2>1
E135	11.9	19.1	44.2	35.7	34.5	32.5	46.3	20.3	21.6	load7>3>4>5>6>9>8>2>1

Dentin										
E0	11.6	12.2	42.7	41.3	25.2	22.8	45.8	15.1	15.0	load7>3>4>5>6>8 = 9>2>1
E90	11.5	16.2	42.9	40.9	33.5	22.4	460	15.9	19.3	load7>3>4>5>6>9>8>2>1
E135	11.9	12.5	43.3	42.9	25.1	22.7	46.4	15.0	15.1	load7>3>4>5>6>8 = 9>2>1

### Effect of different margin designs and loading conditions on stress distribution

**Fig. 5:**
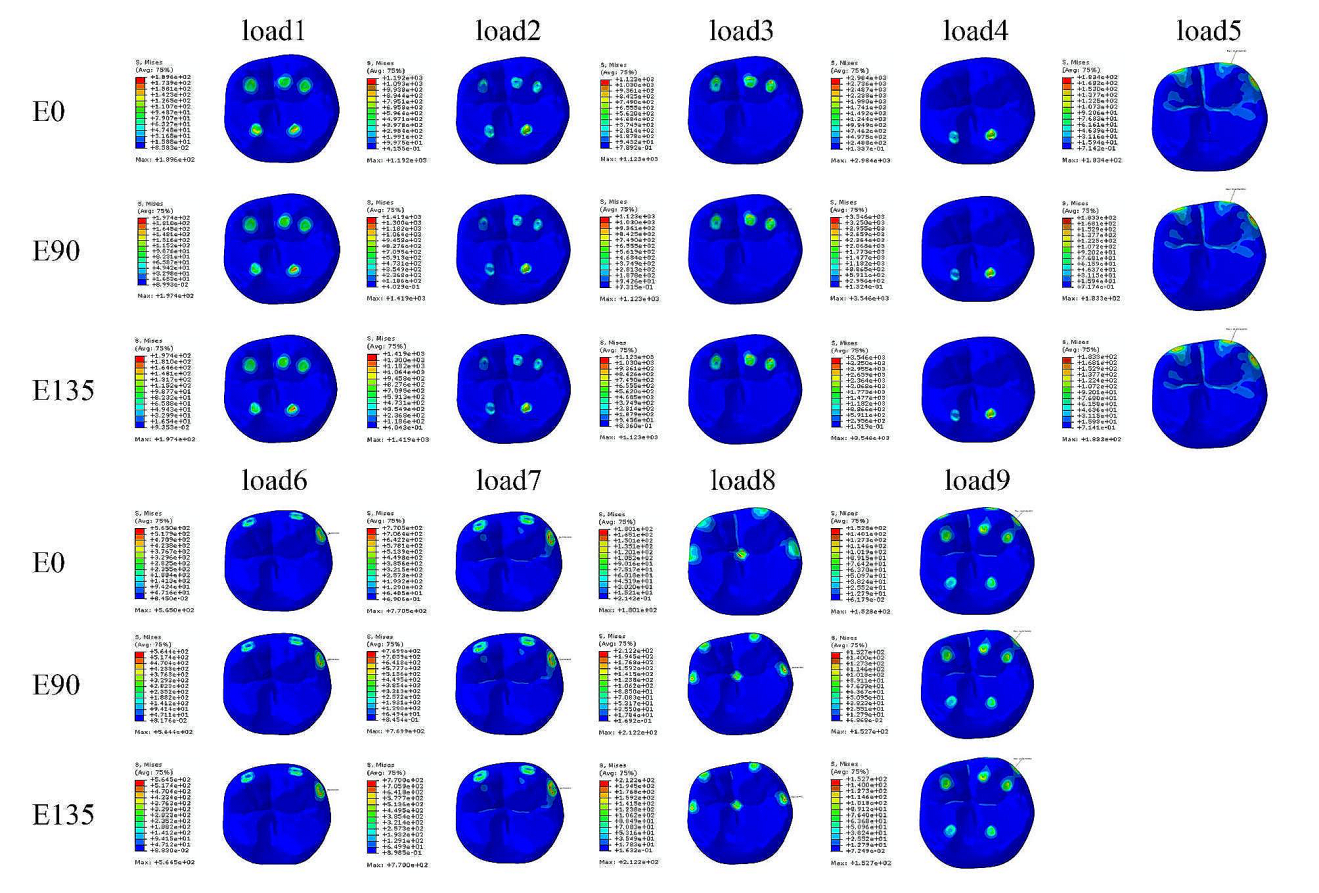
Endocrowns stress distribution cloud diagram of E0, E90 and E135 models under 9 loading conditions. The stresses in the endocrowns were concentrated in the loading area for all models. The scales in the figures showed stresses from high to low in colors. Red color to the blue showed the stresses from high to low

**Fig. 6:**
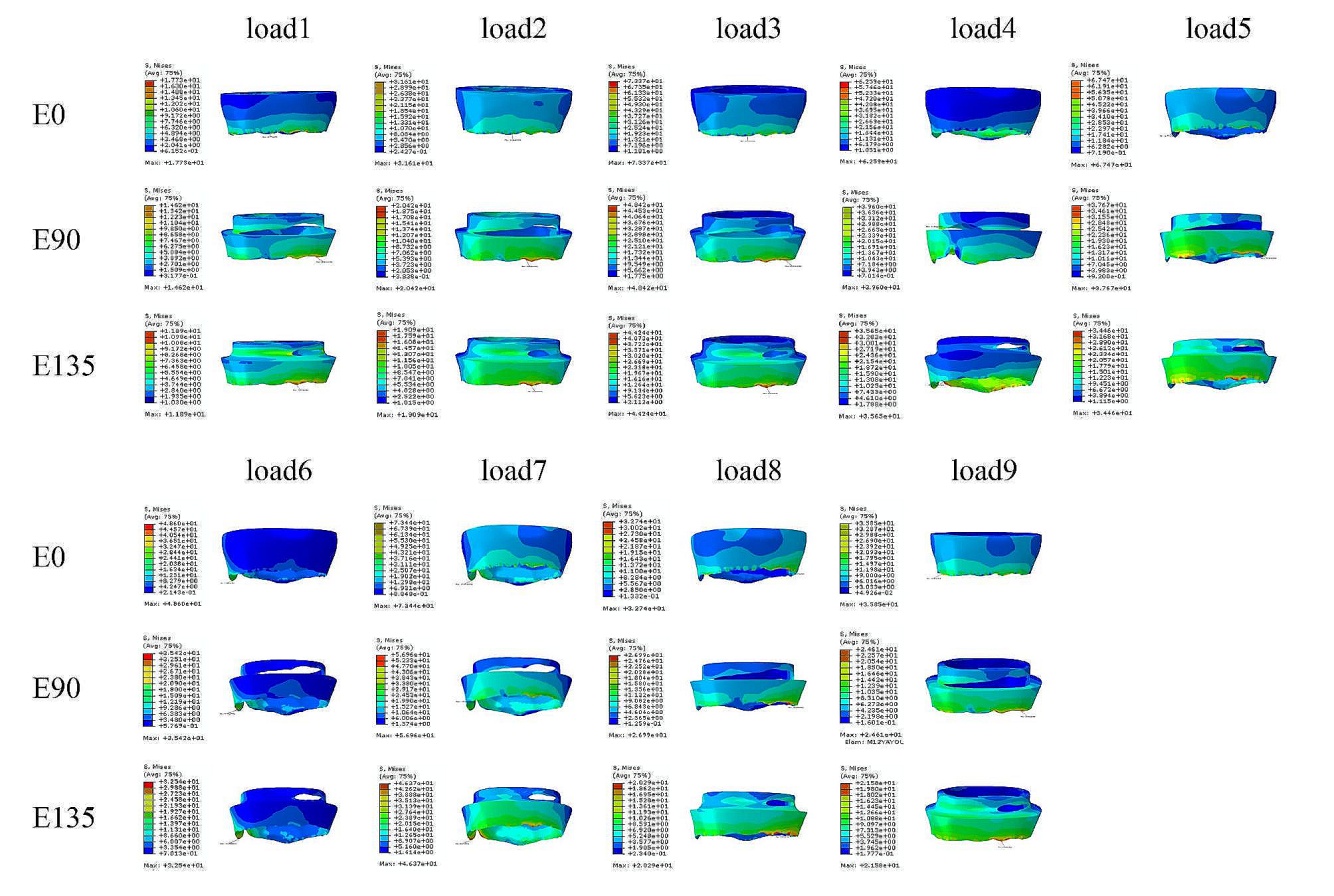
Enamel stress distribution cloud diagram of E0, E90 and E135 models under 9 loading conditions. The stresses of group E0 were concentrated at the cementoenamel junction, while the stresses of group E90 and group E135 were concentrated not only at the cementoenamel junction, but also at the shoulder. In particular, the maximum von Mises stresses of group E90 appeared at the shoulder under load 4. Under the loading conditions of load1, load2, load3, load5, load6, load7, load8 and load9, the stresses of the three groups were concentrated on the buccal side, while under the loading condition of load4, the stresses were concentrated on the lingual side. The scales in the figures showed stresses from high to low in colors. Red color to the blue showed the stresses from high to low

**Fig. 7:**
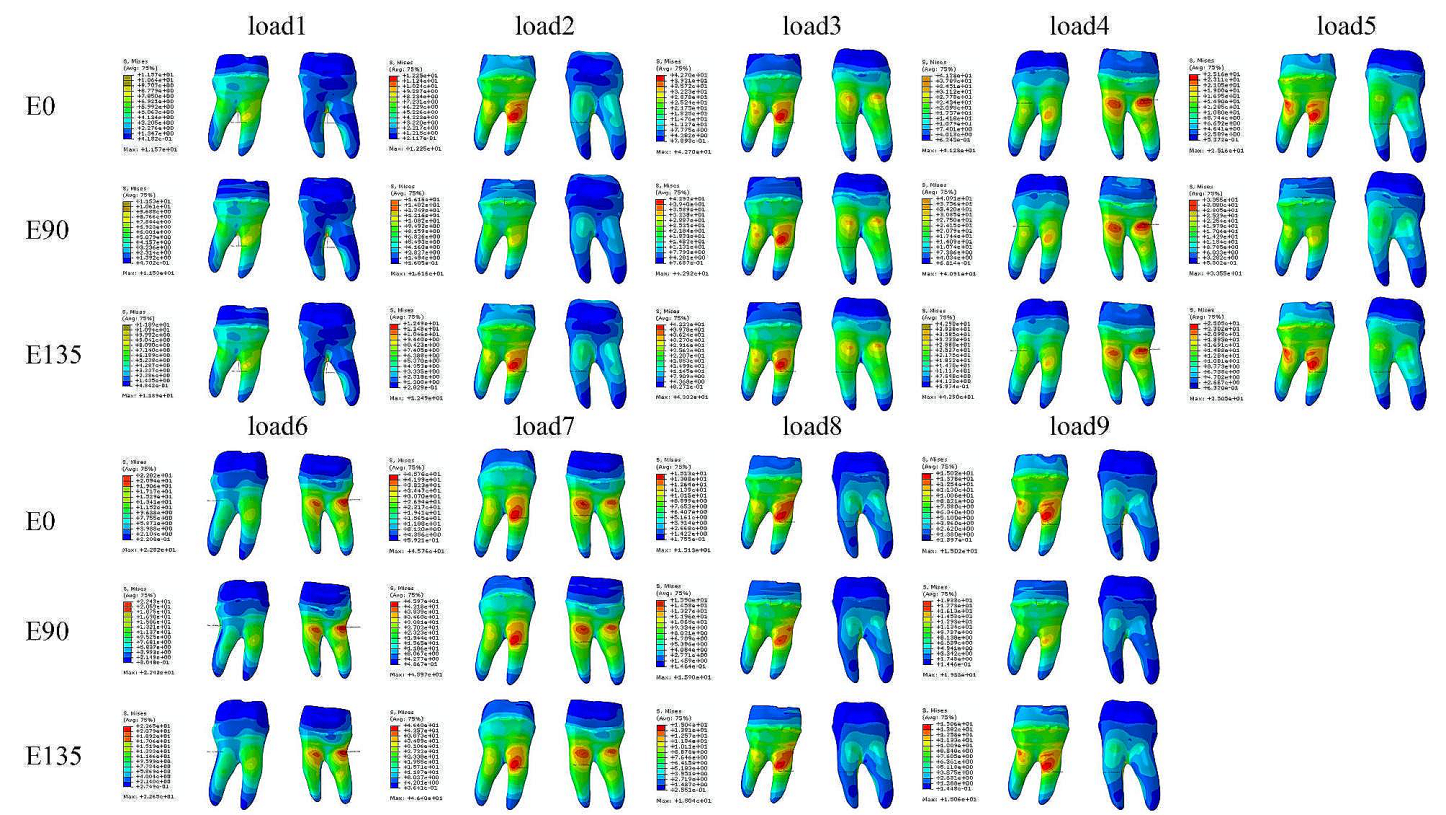
Dentin stress distribution cloud diagram of E0, E90 and E135 models under 9 loading conditions. The stresses were mainly concentrated in the tooth root, especially the upper section of the tooth root. Under the load1, the stresses of the three groups were all concentrated in the root furcation area, and the stresses on the buccal side were higher than that on the lingual side. Under the load2, load3, load5, load8 and load9, the stresses of the three groups of models were all concentrated in the upper section of the tooth root, and the stresses on the buccal side were significantly higher than that on the lingual side. The maximum von Mises stresses of the model E90 appeared at the cementoenamel junction under the load2, load5, load8 and load9. Under the load4 and load6, the stresses of the three groups of models were all concentrated in the upper section of the tooth root, and the stresses on the lingual side were significantly higher than that on the buccal side. Under the load 7, the three groups of models all showed aggregation in the upper section of the buccal and lingual side of the roots, and the buccal side was slightly more obvious than the lingual side. The left was the buccal view and the right was the lingual view. The scales in the figures showed stresses from high to low in colors. Red color to the blue showed the stresses from high to low

The stresses in the endocrowns were concentrated in the loading area in all models, regardless of the margin designs and loading conditions. In the enamel, the stresses of group E0 were concentrated at the cementoenamel junction, while the stresses of group E90 and group E135 were not only concentrated on the cementoenamel junction, but the stresses at the shoulder were significantly higher than that of group E0. In particular, the maximum von Mises stresses of group E90 appeared at the shoulder under load 4 (Fig. 5). The stresses in the enamel of the three model groups were concentrated on the buccal side under the loading conditions of load1, load2, load3, load5, load6, load7, load8 and load9, whereas the stresses were concentrated on the lingual side under the loading condition of load4 (Fig. 6). In dentin, the stresses were mainly concentrated in the tooth root, especially the upper section of the tooth root. The stress concentration areas of the E0 group, E90 group and E135 group were similar under the same loading conditions, but the stress distribution of the models under different loading conditions were quite different. Under the load1 loading condition, the stresses of the three groups of models were all concentrated in the root furcation area, and the stresses on the buccal side were higher than that on the lingual side. Under the loading conditions of load2, load3, load5, load8 and load9, the stresses of the three groups of models were all concentrated in the upper section of the tooth root, and the stresses on the buccal side were significantly higher than that on the lingual side. What was special among them was that the maximum von Mises stresses of the model E90 appeared at the cementoenamel junction under the loading conditions of load2, load5, load8 and load9. Under the loading conditions of load4 and load6, the stresses of the three groups of models were all concentrated in the upper section of the tooth root, and the stresses on the lingual side were significantly higher than that on the buccal side. Under the loading condition of load 7, the three groups of models all showed aggregation in the upper section of the buccal and lingual side of the roots, and the buccal side was slightly more obvious than the lingual side (Fig. 7).

## Discussion

Endocrowns have become increasingly popular in recent years due to its minimally invasive, good adhesion and ease of operation. A large number of studies have reported the excellent clinical results of endocrowns, and they have become the commonly used restoration design solutions by clinicians [4, 28]. In published clinical studies and experimental studies, endocrowns have been proven to have a success rate and fracture resistance similar to or even better than that of full crowns, but endocrowns were more prone to severe tooth tissue fracture, resulting in the inability to repair it again [29, 30]. There were many factors that can affect the stress distribution of tooth tissues, including restoration design, occlusal force distribution, occlusal force direction, occlusal force magnitude, tooth tissue mechanical properties, prism direction at the finishing margin area, pulp chamber extension angles, filling materials, etc [31–34]. . . Appropriate endocrowns designs should balance the stress distribution in all parts of the tooth to avoid excessive local stress concentration, which may lead to complications such as fracture and detachment. In this study, we designed three FEA models of endocrowns: butt-joint type, 90° shoulder type and 135° shoulder type, and simulated the stress distribution under 9 different loading conditions. A more comprehensive evaluation of the effects of different margin designs and loading conditions on endocrowns stress distribution provided more theoretical support for clinical practice.

The influence of margin designs on stress distribution of endocrowns has received much research attention. In this study, three margin design models of butt-joint type, 90° shoulder type and 135° shoulder type were established. Under the same loading conditions, the maximum von Mises stresses of the endocrowns of the three margin design models were basically similar. Only under the loading conditions of load1, load2, load4 and load8, group E0 were less than group E90 and group E135. Compared with other loading conditions, load1, load2 and load4 were all loaded at the lingual cusps. The lingual cusps of the mandibular posterior teeth were non-functional cusp, differences in the shape and inclination angle of the buccal and lingual sides of the teeth may cause differences in stress distribution when the lingual cusps were loaded, but the specific reasons need further study. In addition, the stresses in endocrowns were concentrated in the loading area under all loading conditions. This was because the loading methods used in this study were area loading, and the stresses were concentrated in the loading area and then transferred to other areas. The study by Rocha et al. also found that stresses will be concentrated in the loading area of the restoration [35].

In enamel, regardless of the loading conditions, the maximum von Mises stresses of the three margin design models were E0 > E90 > E135. The margin of group E0 retains thick enamel in contact with the restoration. The difference in Young’s modulus between enamel and dentin allowed the enamel to withstand more force. The shoulder design formed a ferrule that can adequately distribute the force borne by the enamel. Ahmari et al. demonstrated through in vitro mechanical experiments that an appropriate dentin collar can improve the fracture strength of tooth tissue [36]. This study also found that due to the inclined plane design, the 135° shoulder had a more uniform stress distribution compared with the 90° shoulder. Guo et al. also observed results consistent with this study in premolars, with the 135° shoulder design conducted more uniform stresses than the 90° shoulder design [14].

In dentin, the maximum von Mises stresses of the three margin design models were basically similar. In particular, under the loading conditions of load2, load5 and load9, the E90 group model showed stress concentration at the cementoenamel junction, and the maximum von Mises stresses were higher than that of the E0 group and E135 group. The sharp margin contact at the shoulder of the E90 group may be the cause of the cervical stress concentration. zheng et al. analyzed the influence of butt-joint type, bevel type and 90° shoulder type on the stress distribution of mandibular first molar. The results showed that the stresses were mainly concentrated in the upper section of the root. The maximum von Mises stresses of bevel type and 90° shoulder type were lower than that of butt-joint type, which were consistent with the results of this study [17]. The study by AboElhassan et al. found that the stresses in the dentin of the shoulder type were higher than that of the butt-joint type, which may be due to the difference in tooth preparation. AboElhassan’s study was prepared horizontally at 2 mm supragingival to the cementoenamel junction, with less enamel remaining [37], while this study only lowered 1.5 mm of space on the occlusal surface for restoration design, retaining more enamel and dentin, so that more of the stresses were shared by the enamel.

Restorations can be subjected to various occlusal forces in the oral cavity. Evaluating the performance of restorations under different loading conditions can provide a more comprehensive understanding of the advantages and disadvantages of different restoration designs. In this study, 9 loading conditions were designed to evaluate the stresses differentiation of three margin design endocrowns models. In the endocrowns, the maximum von Mises stresses of the three margin design models were load4 > load2 and load3 > load7 and load6 > load8, load1, load5 and load9. The maximum von Mises stresses of load4 were significantly higher than other loading conditions, followed by load2 and load3. These three loading conditions all have lingual loading, the lingual cusps of the mandibular posterior teeth were non-functional cusps, so it can be speculated that the force on the non-functional cusps were more likely to cause stress concentration than the force on the functional cusps. This result may be caused by differences in the shape and inclination angle of the buccal and lingual sides of the teeth. In enamel and dentin, the maximum von Mises stresses of the three margin design models were load7 > load3 and load4 > load5 and load6 > load9 and load8 > load2 > load1. Moreover, the stress concentration areas of the three margin design models were similar under the same loading conditions. In enamel, the stresses were mainly concentrated at the cementoenamel junction, and in dentin, the stresses were mainly concentrated at the tooth root, especially the upper section of the tooth root. Zheng et al. studied the stress distribution of endocrowns with different margin designs under sliding vertical loading conditions. The results showed that all models had stress concentrations in the cementoenamel junction and the tooth root [17]. This is because this area was the junction area between enamel and dentin, the tooth tissue was weak, and it was the fulcrum position of stresses. The maximum von Mises stresses of load7 were significantly higher than other loading conditions, and the three groups of models all have stresses accumulation in the upper section of the buccal and lingual side of the roots. proving that the less the loading angle, the higher the force endured by the tooth tissue and the higher the risk of tooth root fracture and splitting [38]. The maximum von Mises stresses of load3, load4, load5 and load6 were in the middle, and they were all unilateral vertical or inclined loading. The maximum von Mises stresses of load8, load9, load2 and load1 were relatively less because the loading forces were more evenly, proving that uneven force may cause higher harm to the tooth tissue. Under the loading conditions of load2, load5, load8 and load9, the stresses in the dentin of the E90 model were not only concentrated on the tooth root, but also appeared at the cementoenamel junction. The 90° shoulder removed more axial wall dental tissue, and the shoulder was in vertical contact with the endocrowns, resulting in stress concentration in the weak areas of the cervical. Stoilov et al. compared the fatigue resistance of butt–joint type and 90° shoulder type endocrowns, and the results showed that the margin designs had no significant impact on fatigue resistance. However, under very high loads, the marginal area of the ferrule represented a weak point [39]. In addition, there was a correlation between the direction of stress concentration in dentin and the loading position. When loading on the buccal side, stress concentration was likely to occur on the buccal side of the tooth root. When loading on the lingual side, stress concentration was likely to occur on the lingual side of the root. When loading horizontally, significant stress concentration may occur on both the buccal and lingual side of the roots [40].

In this study, only FEA was used to compare the effects of different margin designs and loading conditions on the stress distribution of tooth and endocrowns. The specific choice of margin design should be based on margin suitability, microleakage, flexural strength and long-term clinical follow-up observation. In addition, the choice of margin design form was also affected by factors such as the thickness and height of the tooth axial wall, bonding strength, etc. For example, when the height or width of the remaining tooth tissue wall is insufficient, the butt-joint type margin design can retain more tooth tissue and ensure the strength of the tooth tissue. In addition, when there was less enamel remaining, the shoulder design will remove more enamel, which may affect the bonding strength of endocrowns. More factors need to be considered in the actual application process. Tribst’s research found that endocrowns can be used in 3-unit lithium disilicate fixed partial denture (FPD) [41]. The impact of the margin designs and loading conditions used in this study on endocrown-supported FPD is unclear. In the future, more influencing factors can be included and more groupings can be designed to further improve the experimental evidence. In addition, the connection relationship between the models in the FEA study was a fixed connection, and the materials of the tooth tissue and restoration were a uniform, continuous and isotropic linear elastic, which were different from the actual tooth structure, may cause the conduction and distribution of stresses in the FEA model to be inconsistent with reality. Therefore, more in vitro experiments and clinical trials need to be combined in the future to make a more realistic evaluation.

## Conclusion

In conclusion, while stress distribution is similar among the three margin designs of endocrowns, shoulder designs, especially the 135° shoulder, exhibit reduced stress concentration. This suggests that in clinical practice, shoulder designs may offer advantages in optimizing restoration longevity. Clinicians should consider tooth morphology and axial wall thickness when selecting margin designs, aiming to minimize stress-related complications.

## Data Availability

All data generated or analyzed during this study are included in this published article.
